# Association between receiving information on obstetric complications and institutional delivery: An analysis of the demographic and health survey of Peru, 2019

**DOI:** 10.1016/j.heliyon.2023.e21146

**Published:** 2023-10-26

**Authors:** Carlos Quispe-Vicuña, Daniel Fernandez-Guzman, Brenda Caira-Chuquineyra, Virgilio E. Failoc-Rojas, Guido Bendezu-Quispe, Diego Urrunaga-Pastor

**Affiliations:** a Sociedad Científica San Fernando, Universidad Nacional Mayor de San Marcos, Lima, Peru; b Grupo Peruano de Investigación Epidemiológica, Unidad para la Generación y Síntesis de Evidencias en Salud, Universidad San Ignacio de Loyola, Lima, Peru; c Universidad Científica del Sur, Lima, Peru; d Facultad de Medicina, Universidad Nacional de San Agustín de Arequipa, Arequipa, Peru; e Vicerrectorado de Investigación, Universidad San Ignacio de Loyola, Lima, Peru; f Universidad César Vallejo. Escuela de Medicina, Trujillo, Peru

**Keywords:** Knowledge, Pregnancy complications, Parturition, Home childbirth, Public health, Prenatal education, Peru

## Abstract

**Objective:**

To evaluate the association between receiving information on obstetric complications and institutional delivery in Peruvian women in 2019.

**Methods:**

We conducted a secondary analysis of the 2019 Peruvian Demographic and Family Health Survey (ENDES) database. The dependent variable was the type of delivery (institutional or non-institutional). The exposure variable was self-reporting of having received information on obstetric complications during prenatal care. The association of interest was evaluated using binary logistic regression models, obtaining crude odds ratios (cOR) and adjusted odds ratios (aOR) with their respective 95 % confidence intervals (95%CI). Values of p < 0.05 were considered statistically significant.

**Results:**

We included a total of 14,835 women in the analysis. Of the total, 14,088 (94.1 %) reported having received information on pregnancy complications. Also, 13,883 (92.5 %) had an institutional delivery in their last pregnancy. The adjusted model showed that women who reported knowing the complications that can occur in pregnancy had a higher probability of presenting an institutional delivery (aOR = 1.47; 95%CI: 1.04–2.08).

**Conclusions:**

Receiving information about pregnancy complications was found to be associated with a higher probability of institutional delivery. Ensuring the provision of information to the pregnant woman about pregnancy complications can be a useful strategy to increase institutional delivery.

## 1 Introduction

Maternal mortality has significantly decreased worldwide. According to the World Health Organization (WHO), in 2020 there were 152 deaths per 100,000 live births around the world, and by 2030 this rate is expected to drop further [1]. Maternal mortality is associated with complications during the puerperium, the most frequent being puerperal hemorrhages and infections, and postpartum hypertension [2]. Likewise, in 2020, the United States reported a neonatal mortality rate per 10,000 live births of 13.66 and 27.98 among planned and unplanned home births, respectively [3].

The care of a professional during childbirth, in an appropriate environment and with adequate hygienic conditions can help reduce the risk of complications that can lead to the death of the mother and/or child [4]. Despite this, many national health programs do not adequately promote this care [5,6]. In low- and middle-income countries, factors such as socioeconomic status, the number of prenatal care (PNC) visits, the distance to the nearest hospital, the influence of the media, receiving information during care, and acquiring knowledge about complications associated with childbirth [[7], [8], [9]] can greatly impact maternal health. In Peru, in 2020, maternal mortality was 69t deaths per 100,000 live births [10], which still did not achieve the rate of less than 70 deaths per 100,000, as described in the third Sustainable Development Goal [11].

Knowledge about health risks could induce health-seeking behaviors in pregnant women, as it is described in the literature [12,13]. For this reason, the WHO PNC model recommends providing relevant information and promoting institutional deliver [14]. Few studies reported that women who knew the warning signs and complications of childbirth and were informed about pregnancy complications during their PNC visits were up to two times more likely to have an institutional delivery and to increase birth preparedness (a factor that increases institutional delivery) [[15], [16], [17]]. However, information about this association is scare, and that not necessarily providing this information is associated to the use of maternal health services [18].

In Peru, the Peruvian Ministry of Health in concordance with WHO PNC guidelines, has established that information about pregnancy complications and where to go if they occur is a component of PNC in Peruvian territory [19]. Although in this country the number of institutional deliveries at the national level has been increasing in the last decade [20], this increase has not been similar in each region. Home births, almost entirely attended by traditional midwives, are more frequent in rural areas and are conditioned by social and economic factors such as poverty and education [21,22]. Although maternal health strategies such as PNC aim to provide information on birth complications to promote the continuum of maternal care with trained professionals, including the promotion of institutional delivery, in Peru, there needs to be described if providing this information improves the continuum of attention for childbirth. Hence, the objective of this study was to evaluate the association between receiving information on obstetric complications and institutional delivery in Peruvian women, adjusting for potential confounding variables, to generate useful evidence for health strategies focused on educating pregnant women regarding obstetric complications and increasing institutional delivery.

## 2 Methods

### 2.1 Study design

We conducted a secondary data analysis of the 2019 Peru Demographic and Family Health Survey (ENDES) database. The ENDES is developed annually through the National Institute of Statistics and Informatics (INEI).

The ENDES is representative at the urban-rural, regional, and national levels, as it is a multi-stage survey with a probabilistic sampling design by conglomerates and stratified at the departmental level, as well as by urban and rural areas. The primary sampling unit is made up of selected clusters and the secondary sampling unit was the dwellings in the clusters. Additional information on the ENDES survey methodology is available in its technical report [23].

### 2.2 Population, sample, and sampling

**Fig. 1:**
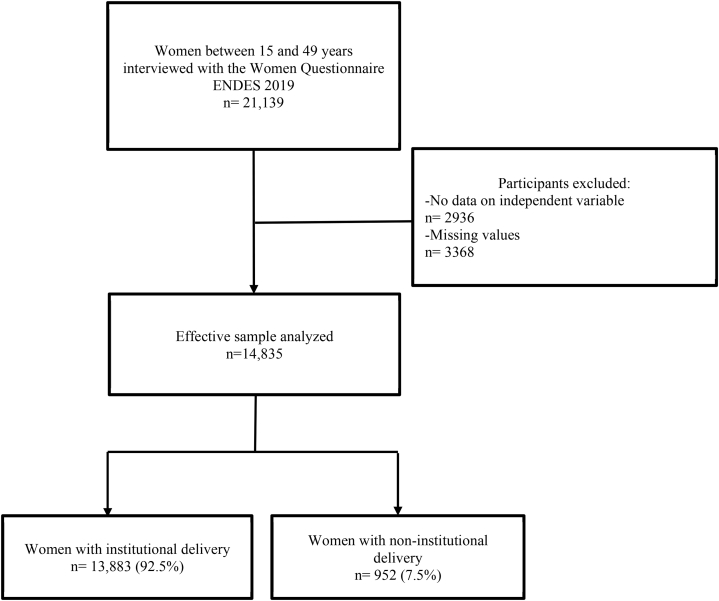
Flowchart of sample selection.

The ENDES collected information from 21,139 women of reproductive age (15–49 years) during 2019. For this study, women of reproductive age who answered the questions that make up the dependent variable (place of delivery), and the independent variable (received information on obstetric complications) and who, in addition, had complete data on the rest of the covariates were included. Women who did not receive antenatal care (ANC) and who were not seen by a trained provider were excluded. The effective sample for our study consisted of 14,835 women (Fig. 1).

### 2.3 Variables

The dependent variable of the study was the place of delivery, collected by self-reporting to the following question "Where did you give birth to (name of each child)?". According to this, the place of delivery was categorized as institutional if the woman referred any of the following answers: 1) Hospital of the Ministry of Health of Peru (MINSA) 2) Hospital of the Social Security of Peru (ESSALUD), 3) Hospital of the armed forces or of the national police of Peru; 4) MINSA health center; 5) MINSA health post; 6) ESSALUD Center/Post; 7) Hospital/other of the municipality; 8) private clinic and 9) clinic/post of a non-governmental organization. Otherwise, it was classified as non-institutional.

The independent variable of the study was the self-reporting of having received information on obstetric complications, which was collected through the question: “Did they explain to you about the complications that can occur in pregnancy?” The response options were: “Yes”, “No” and “Don't know”. The last category was considered as missing data and was excluded.

The following covariates of interest were selected as described in the literature [16]: sociodemographic characteristics such as the mother's age (15–26 years, 27–33 years, 34–49 years), marital status (married or cohabiting, not married or cohabiting), educational level (primary or preschool, secondary, higher), employment status (yes, no), health insurance (yes, no), geographic region (Metropolitan Lima, rest of the coast, highlands, jungle), area of residence (urban, rural), level of wealth (first quintile, second quintile, third quintile, fourth quintile, fifth quintile), ethnicity (mestizo, Quechua, black, others); obstetric characteristics such as parity (first child, second child, third child or more), number of PNC visits (six or more, less than six). Intimate partner violence (IPV) was constructed from questions related to psychological violence (10 items), physical violence (7 items) and sexual violence (2 items) at some point in life. A positive response to any of these three components was considered to categorize a respondent as having IPV.

### 2.4 Statistical analysis

The 2019 ENDES databases were downloaded and imported into the R statistical software program, version 4.1.0 (R Foundation for Statistical Computing, Vienna, Austria), where the databases were merged. The unified database was exported to the Stata® v.17.0 program (Stata Corporation, College Station, Texas, United States) for statistical analyses. All the analyses were conducted considering the complex sampling and the weighting factors of the ENDES using the Stata module svy for complex samples.Since all the variables for the descriptive analysis are categorical, absolute frequencies and weighted proportions were calculated. For the bivariate analysis, the chi-square test with Rao-Scott correction was used to compare the proportions of the covariates of interest with the place of delivery.

To assess the association between receiving information on obstetric complications and non-institutional delivery, binary logistic regression models were used, thus calculating crude (cOR) and adjusted (aOR) odds ratios. For the adjusted analysis, these adjusted variables were included according to epidemiological criteria. The analyses were presented with their respective 95 % confidence intervals (CI) and p values less than 0.05 were considered statistically significant. To assess the collinearity of the variables included in the adjusted model, the variance inflation factor was used, where a value higher than 10 determined multicollinearity between the variables; however, all the values obtained were less than 10.

### 2.5 Ethical aspects

This study did not require the approval of an ethics committee because the database used is freely available on the INEI website (http://iinei.inei.gob.pe/microdatos/). We did not use information that allows the identification of the subjects surveyed, maintaining confidentiality. The primary data collection was carried out by the INEI team, which requested informed consent from the respondents.

## 3 Results

Of a total of 21,139 women of reproductive age between the ages of 15 and 49 who answered the women's questionnaire in ENDES 2019 and who also presented at least one PNC check-up by a trained provider, 2936 women were excluded for not responding to our variable of exposure and 3368 for presenting incomplete information in some of the covariates, obtaining a final sample of 14,835 women (Fig. 1).

### 3.1 General characteristics of the study population

The most frequent age groups were from 27 to 33 years and from 34 to 49 years (both with 35.0 %), the majority were married or cohabiting (89.9 %), and a secondary level of education was the most frequently reported (45.1 %). The largest proportion of participants were from metropolitan Lima (28.8 %), urban areas (73.8 %), and the poorest wealth quintiles (49.3 %). Similarly, most of the women surveyed reported having three or more children (35.7 %) and six or more PNC check-ups (91.8 %) (Table 1).

**Table 1:** Characteristics of the study population (n = 14,835).

Characteristics	n	%^a^	95%CI^a^
**Age**			
15 to 26	4591	30.1	29.1–31.0
27 to 33	5211	35.0	34.0–36.0
34 to 49	5033	35.0	34.0–36.0
**Current marital status**			
Married/cohabiting partner	13,274	89.9	89.2–90.5
Not married/cohabiting partner	1561	10.1	9.5–10.8
**Educational level**			
Primary or preschool	2921	19.2	18.4–20.1
Secondary	6878	45.1	44.0–46.2
Higher	5036	35.7	34.6–36.8
**Employment status**			
Yes	9951	66.1	65.0–67.2
No	4884	33.9	32.8–35.0
**Health insurance**			
Yes	12,388	81.8	80.9–82.7
No	2447	18.2	17.3–19.1
**Geographical region**			
Lima Metropolitan Area	1780	28.8	27.8–29.9
Rest of coastline	4469	26.2	25.1–27.3
Highlands	4955	28.0	26.7–29.3
Jungle	3631	17.0	16.0–18.1
**Area of residence**			
Urban	10,544	73.8	72.9–74.7
Rural	4291	26.2	25.3–27.1
**Wealth index**			
Poorest	8166	49.3	48.1–50.4
Middle	2975	19.7	18.8–20.6
Richest	3694	31.0	29.9–32.2
**Ethnicity**			
Mestizo	6181	45.4	44.3–46.6
Quechua	4270	24.3	23.4–25.2
Black	1578	11.9	11.2–12.6
Others	2806	18.4	17.5–19.4
**Parity**			
First child	4319	29.9	29.0–30.9
Second child	5037	34.4	33.3–35.4
Third child or more	5479	35.7	34.7–36.8
**Number or PNC visits**			
≥6	13,621	91.8	91.2–92.4
<6	1214	8.2	7.6–8.8
**Intimate partner violence**			
No	6884	46.6	45.5–47.7
Yes	7951	53.4	52.3–54.5
**Know the complications that can occur in pregnancy**			
No	747	5.9	5.4–6.5
Yes	14,088	94.1	93.5–94.6
**Type of delivery**			
Non-institutional	952	7.5	6.8–8.2
Institutional	13,883	92.5	91.8–93.2

PNC: prenatal care; CI: confidence interval.

a Weighted percentages according to survey complex sampling.

### 3.2 Characteristics of the study population according to the place of delivery

The prevalence of institutional delivery was 92.5 %, being higher in women not married or cohabiting (94.6 %; p = 0.007), those with a higher level of education (96.4 %; p < 0.001) and in women with health insurance (93.0 %; p = 0.001). In addition, a higher proportion of institutional delivery was observed in women residing in metropolitan Lima (95.8 %, p < 0.001), in an urban area (96.2 %; p < 0.001), those in the highest wealth indices (97.4 %; p < 0.001), women who belonged to the Quechua ethnic group (96.2 %; p < 0.001), who had their first child (94.5 %; p < 0.001), who had six or more PNC check-ups (93 %; p < 0.001), and those who suffered from IPV (93.2 %; p = 0.015) (Table 2).

**Table 2:** Prevalence of non-institutional delivery according to the characteristics of the study population (n = 14,835).

Characteristics	Type of delivery						
	Non-institutional			Institutional			*p-value*^b^
	n	%^a^	95%CI^a^	n	%^a^	95%CI^a^	
**Age**							
15 to 26	328	8.6	7.5–9.8	4263	91.4	90.2–92.5	0.052
27 to 33	315	7.1	6.2–8.1	4896	92.9	91.9–93.8	
34 to 49	309	7.0	6.0–8.1	4724	93.0	91.9–94.0	
**Current marital status**							
Married/cohabiting partner	878	7.7	7.0–8.5	12,396	92.3	91.5–93.0	**0.007**
Not married/cohabiting partner	74	5.4	4.1–6.9	1487	94.6	93.1–95.9	
**Educational level**							
Primary or preschool	487	19.0	16.9–21.4	2434	81.0	78.6–83.1	<**0.001**
Secondary	334	5.7	4.9–6.5	654	94.3	93.5–95.1	
Higher	131	3.6	2.8–4.6	4905	96.4	95.4–97.2	
**Employment status**							
Yes	598	7.1	6.3–7.9	9353	93.0	92.1–93.7	0.055
No	354	8.4	7.2–9.7	4530	91.6	90.3–92.8	
**Health insurance**							
Yes	756	7.0	6.3–7.8	11,632	93.0	92.2–93.7	**0.001**
No	196	9.9	8.2–11.7	2251	90.1	88.3–91.8	
**Geographical region**							
Lima Metropolitan Area	67	4.2	3.2–5.5	1713	95.8	94.5–96.8	<**0.001**
Rest of coastline	185	5.2	4.3–6.4	4284	94.8	93.6–95.7	
Highlands	343	9.3	7.9–10.9	4612	90.7	89.1–92.1	
Jungle	357	13.8	11.7–16.2	3274	86.2	83.8–88.3	
**Area of residence**							
Urban	315	3.8	3.3–4.4	10,229	96.2	95.6–96.7	<**0.001**
Rural	637	18.0	15.9–20.3	3654	82.0	79.7–84.1	
**Wealth index**							
Poorest	777	12.0	10.8–13.3	7389	88.0	86.7–89.2	<**0.001**
Middle	96	4.1	3.1–5.4	2879	95.9	94.6–96.9	
Richest	79	2.6	1.9–3.4	3615	97.4	96.6–98.1	
**Ethnicity**							
Mestizo	277	5.1	4.4–5.8	5904	94.9	94.2–95.6	<**0.001**
Quechua	156	3.8	3.0–4.9	4114	96.2	95.1–97.0	
Black	159	12.1	10.0–14.5	1419	87.9	85.5–90.0	
Others	360	15.5	13.3–17.9	2446	84.6	82.1–86.7	
**Parity**							
First child	183	5.5	4.6–6.6	4136	94.5	93.4–95.4	<**0.001**
Second child	246	5.8	5.0–6.8	4791	94.2	93.2–95.0	
Third child or more	523	10.8	9.6–12.1	4956	89.2	87.9–90.4	
**Number or PNC visits**							
≥6	802	7.0	6.3–7.7	12,819	93.0	92.3–93.7	<**0.001**
<6	150	13.0	10.9–15.5	1064	87.0	84.5–89.1	
**Intimate partner violence**							
No	479	8.3	7.3–9.4	6405	91.7	90.6–92.7	**0.015**
Yes	473	6.8	6.1–7.7	7478	93.2	92.3–93.9	
**Know the complications that can occur in pregnancy**							
No	68	9.0	6.7–12.0	679	91.0	88.0–93.3	**0.204**
Yes	884	7.4	6.7–8.2	13,883	92.6	91.8–93.3	

PNC: prenatal care; CI: confidence interval.

a Weighted percentages according to survey complex sampling.

b Calculated by Chi2 test of independence with Rao Scott correction for complex sampling. p-values <0.05 are in bold.

### 3.3 Characteristics of the study population according to the report of having received information on pregnancy complications

The prevalence of having received information about pregnancy complications was 94.1 % with a significantly higher frequency in the age group of 15–26 years (94.7 %; p = 0.026), among women with a secondary education level (94.8 %; p = 0.024), among those residing in the jungle region of Peru (96.6 %; p < 0.001), women who lived in rural areas (95.3 %; p = 0.007), as well as those belonging to the poorest wealth quintiles (95.3 %; p < 0.001), women who received six or more PNC check-ups (94.4 %; p < 0.001), and those who did not present IPV (94.9 %; p = 0.004) (Table 3).

**Table 3:** Prevalence of knowledge of complications according to the characteristics of the study population (n = 14,835).

Characteristics	Know the complications that can occur in pregnancy						
	No			Yes			*p-value*^b^
	n	%^a^	95%CI^a^	n	%^a^	95%CI^a^	
**Age**							
15 to 26	223	5.3	4.5–6.2	4368	94.7	93.8–95.5	**0.026**
27 to 33	239	5.5	4.7–6.5	4972	94.5	93.5–95.3	
34 to 49	285	6.9	6.0–8.0	4748	93.1	92.0–94.0	
**Current marital status**							
Married/cohabiting partner	671	5.9	5.4–6.5	12,603	94.1	93.5–94.6	0.832
Not married/cohabiting partner	76	6.1	4.6–8.1	1485	93.9	91.9–95.4	
**Educational level**							
Primary or preschool	157	6.1	5.0–7.4	2764	93.9	92.6–95.0	**0.024**
Secondary	304	5.2	4.5–6.0	6574	94.8	94.0–95.5	
Higher	286	6.8	5.9–7.9	4750	93.2	92.1–94.1	
**Employment status**							
Yes	513.0	6.1	5.5–6.8	9438	93.9	93.1–94.5	0.364
No	234.0	5.6	4.7–6.6	4650	94.4	93.4–95.3	
**Health insurance**							
Yes	603	5.7	5.1–6.3	11,785	94.3	93.7–94.9	0.055
No	144	7.1	5.8–8.6	14,088	92.9	91.4–94.2	
**Geographical region**							
Lima Metropolitan Area	155	8.6	7.3–10.1	1625	91.4	89.9–92.7	<**0.001**
Rest of coastline	238	5.9	5.0–7.0	4231	94.1	93.0–95.0	
Highlands	211	4.8	4.1–5.6	4744	95.2	94.4–95.9	
Jungle	143	3.4	2.7–4.3	3488	96.6	95.7–97.3	
**Area of residence**							
Urban	565	6.4	5.7–7.1	9979	93.6	92.9–94.3	**0.007**
Rural	182	4.8	3.9–5.7	4109	95.3	94.3–96.1	
**Wealth index**							
Poorest	345	4.7	4.1–5.4	7821	95.3	94.6–95.9	<**0.001**
Middle	171	6.2	5.2–7.6	2804	93.8	92.4–94.8	
Richest	231	7.7	6.6–9.0	3463	92.3	91.0–93.4	
**Ethnicity**							
Mestizo	284	5.6	4.8–6.5	5897	94.4	93.5–95.2	0.205
Quechua	197	5.8	48.6–69.5	4073	94.2	93.0–95.1	
Black	83	5.6	4.3–7.3	1495	94.4	92.6–95.7	
Others	183	7.2	5.9–8.6	2623	92.8	91.4–94.1	
**Parity**							
First child	225	6.1	5.1–7.2	4094	93.9	92.8–94.9	0.439
Second child	266	6.3	5.4–7.3	4771	93.7	92.7–94.6	
Third child or more	256	5.5	4.7–6.4	5223	94.5	93.6–95.3	
**Number or PNC visits**							
≥6	644	5.6	5.1–6.2	12,977	94.4	93.8–94.9	<**0.001**
<6	103	9.2	7.3–11.7	1111	90.8	88.3–92.7	
**Intimate partner violence**							
No	303	5.1	4.4–5.9	6581	94.9	94.1–95.6	**0.004**
Yes	444	6.7	5.9–7.5	7507	93.3	92.5–94.1	
**Type of delivery**							
Non-institutional	68	7.1	5.3–9.5	884	92.9	90.5–94.7	**0.204**
Institutional	679	5.8	5.3–6.4	13,204	94.2	93.6–94.7	

PNC: prenatal care; CI: confidence interval.

PNC visits.

a Weighted percentages according to survey complex sampling.

b Calculated by Chi2 test of independence with Rao Scott correction for complex sampling. p-values <0.05 are in bold.

### 3.4 Association between receiving information about complications in pregnancy and institutional delivery

Regarding the results of the regression models, the crude model found that having received information about pregnancy complications increased the possibility of concluding in an institutional delivery by 1.24 times compared to women who did not receive such information, although the result was not statistically significant (cOR = 1.24; 95%CI: 0.89–1.72). The result of the model adjusted for age, marital status, occupation, wealth, residence, parity, IPV, and number of PNC check-ups, showed that the association maintained the direction and presented statistical significance (aOR = 1.47; 95%CI: 1.04–2.08) (Table 4). Results for the crude and adjusted models for covariates are available in supplementary material (Table S1).

**Table 4:** Association between knowledge of complications and institutional delivery, ENDES 2019.

Characteristics	Crude Model			Adjusted Model		
	cOR	95%CI	p-value	aOR	95%CI	p-value
Know the complications that can occur in pregnancy						
No	Ref.			Ref.		
Yes	1.24	0.89–1.72	0.205	1.47	1.04–2.08	**0.028**

cOR: crude odds ratio; aOR: adjusted odds ratio; CI: confidence interval.

Odds ratios and confidence intervals were calculated considering the survey complex sampling. p-values <0.05 are in bold.

Epidemiological model, adjusted for age, marital status, occupation, wealth, residence, parity, intimate partner violence, and number of prenatal check-ups.

## 4 Discussion

### 4.1 Main findings

The development of complications and death is favored in puerperal women by not having received an institutional delivery. For this reason, in this study national data was analyzed to determine the possible association between receiving information on obstetric complications and institutional delivery in Peruvian women. It was found that institutional delivery is associated with receiving information on obstetric complications during PNC, with institutional delivery being more frequent in women who had received information on obstetric complications compared to those who had not.

### 4.2 Frequency of having received information about obstetric complications

We found that in Peru during 2019 the prevalence of having received some explanation about obstetric complications was 94.1 %. In this regard, it should be noted that the national proportion has improved compared to 2014, increasing by around 2 % [23].

This prevalence was higher than that reported in Bangladesh, which was 46.7 % in 2014 [16]. This difference could be explained by differences in PNC policies and sociocultural behaviors in the two countries. It is important to bear in mind that receiving some explanation about obstetric complications does not guarantee an understanding of this information and therefore does not represent the knowledge of the respondents. Previous studies have reported low frequencies of knowledge of signs of complications, ranging from 15 % to 70 % in populations without educational interventions [[24], [25], [26], [27], [28], [29]], and up to 80.9 % when some educational intervention was performed, with complications of pregnancy being the most frequently known compared to complications of childbirth and the puerperium [30]. For this reason, although the proportion of having received information on obstetric complications in the present study was high, the level of knowledge about danger signs or pregnancy complications in the respondents could not be ascertained. However, greater coverage of this component of PNC may favor institutional delivery, requiring future research on the level of knowledge and place of delivery care in the Peruvian population.

### 4.3 Institutional delivery frequency

We found that in 2019 nine out of ten women of reproductive age had an institutional delivery in Peru. This finding is similar to the 96 % of institutional deliveries described in a previous study in rural India [31]. Likewise, in Peru during the years 2015–2017, a prevalence of non-home delivery of 93 % was reported [32]. This is much more frequent than that described in previous studies carried out in different African countries, in which prevalences between 60.8 % and 77.9 % were found [[33], [34], [35]]. Likewise, a systematic review reported a pooled prevalence of institutional delivery of 31 % in Ethiopia [36] and a previous study in rural Haiti reported a prevalence of 45.4 % [37]. The prevalence of institutional delivery found in this study could be explained by the greater promotion of hospital care for deliveries as well as the universal coverage of health insurance for delivery care for all Peruvian women, which reduces economic and social inequities. for access to institutional delivery [38].

### 4.4 Association of interest

In our study, performing an institutional delivery was 47 % more frequent in women who received information on obstetric complications compared to those who did not. This is in line with previous studies that found an association between these two variables. Ameyaw et al. [16] and Seid et al. [29] found that women who were informed about pregnancy complications during antenatal care were 44 % more likely to perform an institutional delivery compared with those who those who were not informed. Both studies used national demographic and health surveys from Bangladesh in 2014 and Ethiopia in 2016, respectively. Similarly, other studies in low- and middle-income countries such as Tanzania [39], Ethiopia [40], and Eritrea [41] reported that providing health information during antenatal checkups to women was associated with more institutional deliveries. Regarding knowledge about warning signs, the literature describes that women with knowledge about danger signs were more likely to be prepared for childbirth, either having identified the health facility, means of transportation, or saving money for the delivery [15,42,43], thereby increasing institutional delivery care. A possible explanation for this is that women who receive more information about pregnancy complications are more fearful, which motivates them to deliver in a health facility in order to avoid a serious condition [44].

### 4.5 Public health implications

The main contribution of our results in the Peruvian context is to provide a broader overview of the factors that condition a greater use of medical care in childbirth, in addition to promoting counseling regarding obstetric complications during PNC. In Peru, the technical standards established by the MINSA state that during PNC, not only must medical management be provided to the patient, but also information on her condition and possible complications of childbirth [38]. However, it has been reported that in rural areas such as the highlands there is a low quality of PNC [45], which would complicate reporting on delivery complications. Therefore, more specific strategies should be carried out according to the traditional conditions and practices of childbirth in the area [46] similar to what has been done in countries such as Bangladesh [47]. In addition, the quality of care in health centers should also be improved [48] in order to establish a better doctor-patient relationship and provide better information during PNC of pregnant women.

### 4.6 Limitations and strengths

This study has some limitations. First, because it is a secondary analysis study, it is possible that some important confounding variables have not been measured by the ENDES. Second, the design used by the ENDES does not allow evaluating the causality between institutional delivery and the acquisition of knowledge. Third, there may have been a recall bias or inadequate understanding of the questions in some subgroups of women surveyed. Despite these limitations, we believe that the findings of this study can provide an overview of this problem. In addition, it must be borne in mind that the ENDES allows an annual analysis of the national and regional panorama and, also, has methodological quality controls conducted by experts and, therefore, allows evaluation of the general state of health of the Peruvian population.

## 5 Conclusions

The prevalence of institutional deliveries in Peru in 2019 was 92.5 %, which indicates that approximately nine out of ten postpartum women between the ages of 15 and 49 went to a health institution to perform their delivery. Similarly, the frequency of having received information about obstetric complications was 94.1 %, which indicates that approximately nine out of ten women received some explanation from health personnel about possible complications of their delivery. In turn, we found that it was more likely for women who received explanations about possible complications to receive institutional delivery care. The results of this study indicate the need to strengthen and implement information strategies and campaigns on pregnancy complications during PNC to increase delivery care by qualified professionals in an adequate care setting.

## Funding

The study was self-funded. The article processing charge was funded by the Universidad César Vallejo.

## Data availability statement

The database used is freely available on the INEI website (http://iinei.inei.gob.pe/microdatos/).

## CRediT authorship contribution statement

**Carlos Quispe-Vicuña:** Conceptualization, Methodology, Writing – original draft. **Daniel Fernandez-Guzman:** Data curation, Formal analysis, Methodology, Software, Writing – original draft, Writing – review & editing. **Brenda Caira-Chuquineyra:** Data curation, Formal analysis, Methodology, Software, Writing – original draft, Writing – review & editing. **Virgilio E. Failoc-Rojas:** Methodology, Writing – original draft, Writing – review & editing. **Guido Bendezu-Quispe:** Methodology, Writing – original draft, Writing – review & editing. **Diego Urrunaga-Pastor:** Data curation, Formal analysis, Methodology, Writing – review & editing.

## Declaration of competing interest

The authors declare that they have no known competing financial interests or personal relationships that could have appeared to influence the work reported in this paper.
