# Supplementary material for: Association between receiving information on obstetric complications and institutional delivery: An analysis of the demographic and health survey of Peru, 2019
Source: Heliyon. 2023 Oct 26;9(11):e21146. doi: 10.1016/j.heliyon.2023.e21146 (PMC10665671; doi:10.1016/j.heliyon.2023.e21146)
Supplement: Multimedia component 1 [file mmc1.docx]

| **Supplementary material**  **Table S1. Association between knowledge of complications and institutional delivery, ENDES 2019 (including crude and adjusted models for covariates).** | | | | | | | |
| --- | --- | --- | --- | --- | --- | --- | --- |
| **Characteristics** | **Crude Model** | | |  | **Adjusted Model** | | |
|  | **cOR** | **95%CI** | **p-value** |  | **aOR** | **95%CI** | **p-value** |
| **Know the complications that can occur in pregnancy** | | | | | | |  |
| No | Ref. |  |  |  | Ref. |  |  |
| Yes | 1.24 | 0.89 - 1.72 | 0.205 |  | 1.47 | 1.04 - 2.08 | **0.028** |
| **Age** |  |  |  |  |  |  |  |
| 15 to 26 | Ref. |  |  |  | Ref. |  |  |
| 27 to 33 | 1.23 | 1.02 - 1.49 | 0.034 |  | 1.26 | 0.99 - 1.60 | 0.066 |
| 34 to 49 | 1.24 | 1.02 - 1.52 | 0.033 |  | 1.44 | 1.11 - 1.86 | 0.006 |
| **Current marital status** | |  |  |  |  |  |  |
| Married/cohabiting partner | Ref. |  |  |  | Ref. |  |  |
| Not married/ cohabiting partner | 1.48 | 1.11 - 1.98 | 0.008 |  | 1.16 | 0.86 - 1.57 | 0.332 |
| **Educational level** | |  |  |  |  |  |  |
| Primary or preschool | Ref. |  |  |  |  |  |  |
| Secondary | 3.91 | 3.25 - 4.70 | <0.001 |  |  |  |  |
| Higher | 6.26 | 4.68 - 8.36 | <0.001 |  |  |  |  |
| **Employment status** | |  |  |  |  |  |  |
| Yes | Ref. |  |  |  | Ref. |  |  |
| No | 0.83 | 0.68 - 1.00 | 0.055 |  | 0.93 | 0.76 - 1.13 | 0.445 |
| **Health insurance** | |  |  |  |  |  |  |
| Yes | Ref. |  |  |  |  |  |  |
| No | 0.69 | 0.55 - 0.85 | 0.001 |  |  |  |  |
| **Geographical region** | |  |  |  |  |  |  |
| Lima Metropolitan Area | Ref. |  |  |  |  |  |  |
| Rest of coastline | 0.78 | 0.55 - 1.12 | 0.176 |  |  |  |  |
| Highlands | 0.42 | 0.30 - 0.59 | <0.001 |  |  |  |  |
| Jungle | 0.27 | 0.19 - 0.38 | <0.001 |  |  |  |  |
| **Area of residence** | |  |  |  |  |  |  |
| Urban | Ref. |  |  |  | Ref. |  |  |
| Rural | 0.18 | 0.15 - 0.22 | <0.001 |  | 0.27 | 0.22 - 0.34 | <0.001 |
| **Wealth index** |  |  |  |  |  |  |  |
| Poorest | Ref. |  |  |  | Ref. |  |  |
| Middle | 3.19 | 2.35 - 4.33 | <0.001 |  | 1.46 | 1.07 - 1.99 | 0.016 |
| Richest | 5.12 | 3.73 - 7.04 | <0.001 |  | 1.98 | 1.40 - 2.79 | <0.001 |
| **Ethnicity** |  |  |  |  |  |  |  |
| Mestizo | Ref. |  |  |  |  |  |  |
| Quechua | 1.35 | 1.01 - 1.80 | 0.046 |  |  |  |  |
| Black | 0.39 | 0.31 - 0.49 | <0.001 |  |  |  |  |
| Others | 0.29 | 0.23 - 0.37 | <0.001 |  |  |  |  |
| **Parity** |  |  |  |  |  |  |  |
| First child | Ref. |  |  |  | Ref. |  |  |
| Second child | 0.94 | 0.74 - 1.19 | 0.609 |  | 0.86 | 0.66 - 1.12 | 0.255 |
| Third child or more | 0.48 | 0.38 - 0.60 | <0.001 |  | 0.5 | 0.37 - 0.66 | <0.001 |
| **Number or PNC visits** | |  |  |  |  |  |  |
| >=6 | Ref. |  |  |  | Ref. |  |  |
| <6 | 0.50 | 0.41 - 0.62 | <0.001 |  | 0.57 | 0.46 - 0.71 | <0.001 |
| **Intimate partner violence** | |  |  |  |  |  |  |
| No | Ref. |  |  |  | Ref. |  |  |
| Yes | 1.23 | 1.04 - 1.45 | 0.015 |  | 1.27 | 1.06 - 1.50 | 0.008 |
| cOR: crude odds ratio; aOR: adjusted odds ratio; CI: confidence interval. | | | | | | | |
| Odds ratios and confidence intervals were calculated considering the survey complex sampling. p-values <0.05 are in bold. | | | | | | | |
